# Peri‐parturient ewe mortality in commercial, southern Australian, non‐Merino ewe flocks: establishing the frequency and investigating causes

**DOI:** 10.1111/avj.13380

**Published:** 2024-10-07

**Authors:** MC McQuillan, E Glanville, C Jacobson, L Sherriff, DM McGill, A Whale, MB Allworth

**Affiliations:** ^1^ Fred Morley Centre, School of Agricultural, Environmental and Veterinary Sciences Charles Sturt University Wagga Wagga New South Wales 2678 Australia; ^2^ Gubali Centre Charles Sturt University Wagga Wagga New South Wales 2678 Australia; ^3^ Faculty of Veterinary and Agricultural Sciences University of Melbourne Melbourne Victoria Australia; ^4^ Centre for Animal Production and Health Murdoch University Murdoch Western Australia 6150 Australia; ^5^ Pinion Advisory 25 York Street Launceston Tasmania 7250 Australia; ^6^ Livestock Logic 60 Portland Road Hamilton Victoria 3300 Australia; ^7^ Present address: School of Animal and Veterinary Sciences The University of Adelaide Roseworthy South Australia Australia

**Keywords:** ewe, mortality, non‐Merino, peri‐parturient, survival

## Abstract

**Background:**

The level and cause of ewe mortality over the peri‐parturient period is poorly understood in Australia. The purpose of this study was to determine the frequency of peri‐parturient ewe mortality and investigate the causes of death in commercial, non‐Merino ewes over the peri‐parturient period.

**Methods:**

An observational study involving 50 commercial, non‐Merino farms across southern Australia during two lambing seasons was conducted. The study population was the breeding flock of ewes on each farm. Ewes were monitored by farmers from the time they were first placed in their lambing paddocks before lambing, up until lamb marking (the lambing period). The project required no change to normal practice. Veterinarians conducted postmortem (PM) examinations at three time points on each farm over the lambing period. A standard PM protocol was followed by all participating veterinarians.

**Results:**

The mean peri‐parturient mortality over the lambing period was 2.5% in Year 1 and 2.0% in Year 2, with no significant difference between years. Factors that increased the risk of peri‐parturient ewe mortality included ewe age (>5 years old) and litter size (triplet‐bearing ewes). The most common causes of ewe death according to farmers was dystocia and unknown causes. The three most common diagnoses on veterinary PM examination were dystocia, septicaemia and trauma.

**Conclusions:**

This study reveals the relative importance of each cause of ewe mortality over the peri‐parturient period. Risk reduction could include identification and management of older ewes (aged above 5 years or older) and ewes carrying twins or triplets.

**Abbreviations**
AEC: animal ethics committee
BCS: body condition score
BHB: beta hydroxybutyrate
Ca: calcium
FOO: feed on offer
ICT: anthrax immunochromatographic antigen detection assay
LTEM: lifetime ewe management
Mg: magnesium
MLA: meat and livestock Australia
PPT: primary pregnancy toxaemia
PM: postmortem
SDI: significant disease investigation
SPT: secondary pregnancy toxaemia
TSE: transmissible spongiform encephalitis surveillance program
WEC: worm egg count

Australia is the world's largest exporter of sheep meat and is the world's second largest producer of lamb and mutton.^1^ Although lamb losses at and immediately after lambing are a significant contributor to reproductive wastage in the Australian sheep industry,^2^ there is little information on the prevalence and causes of ewe losses in this peri‐parturient period either in Australia or worldwide.^3^ Annual ewe mortality in Australia has been estimated at 2%–11%.^4^, ^5^, ^6^, ^7^, ^8^ These estimates do not delineate what time of year/stage of the reproductive cycle the ewe deaths are most likely to occur. The peri‐parturient period is considered a time of increased mortality risk for ewes.^9^

Risk factors suggested for ewe mortality include multiple‐bearing ewes,^6^, ^8^, ^10^, ^11^ low body weight/fat score and high intestinal parasite burdens^5^ but it is not clear if the latter two factors are pertinent during the peri‐parturient period. Recently, in New Zealand, it has been determined that poor body condition score (BCS) in the peri‐parturient period is a risk factor for ewe mortality.^11^

Causes of peri‐parturient ewe mortality have been classified into three categories: prepartum, during parturition and immediately postpartum.^9^ Conditions causing mortality include metabolic disturbances (prepartum), obstetrical issues (during parturition), clostridial diseases and septicaemia‐toxaemia (pre‐ and postpartum).^9^ It has been noted that dystocia, uterine prolapse, retained placenta and postpartum metritis are the most common obstetrical issues that affect the subsequent fertility of ewes.^12^ There is little evidence in the literature on the prevalence of these diseases and the incidence of ewe mortality associated with these conditions.

Non‐Merino ewes were selected exclusively for this study to allow for more tailored insights into management practices specific to these breeds, which differ significantly from those typically applied to Merino ewes. Bates et al. (2023) highlighted that management practices, including nutritional and reproductive strategies, often vary significantly between Merino and non‐Merino breeds, which can influence outcomes such as ewe mortality during the peri‐parturient period. For example, non‐Merino breeds, such as Composite and shedding breeds, have different nutritional requirements and management practices that can impact their health and reproductive success.^13^ By focusing on non‐Merino flocks, the study aims to provide more relevant data that can be directly applied to improving management strategies for these breeds, thereby enhancing productivity and welfare outcomes.

Furthermore, Merino ewes have distinct physiological and reproductive characteristics that could introduce additional variability into the study outcomes.^13^ By excluding Merino ewes, the research seeks to reduce genetic and management‐related biases, ensuring that the findings are specific to non‐Merino breeds, which are increasingly important in the context of Australia's diverse sheep industry.

The aim of this study was to determine the prevalence of peri‐parturient ewe mortality in commercial, non‐Merino flocks in southern Australia and to determine the causes of these deaths as determined by farmer observation and by veterinary postmortem (PM) examination.

## Methods

### Study design

An observational, cross‐sectional study was conducted over 2 years—2019 (Year 1) and 2020 (Year 2). The target population comprised commercial, non‐Merino ewes on farms in southern Australia during the peri‐parturient period. The farms were chosen based on an open call for eligible farms who fulfilled the following criteria:

1. Located in either Victoria (VIC), New South Wales (NSW), South Australia (SA) or Western Australia (WA).
2. Flock size >500 non‐Merino ewes.
3. An assessment by researchers that a participant had the ability to accurately record ewe deaths during the lambing period.
4. For Vic, NSW and SA, flocks were enrolled that had preferential management of multiple‐bearing ewes from ultrasound pregnancy scanning to lambing as per Lifetime Ewe Management (LTEM) guidelines.^14^

**Figure 1:**
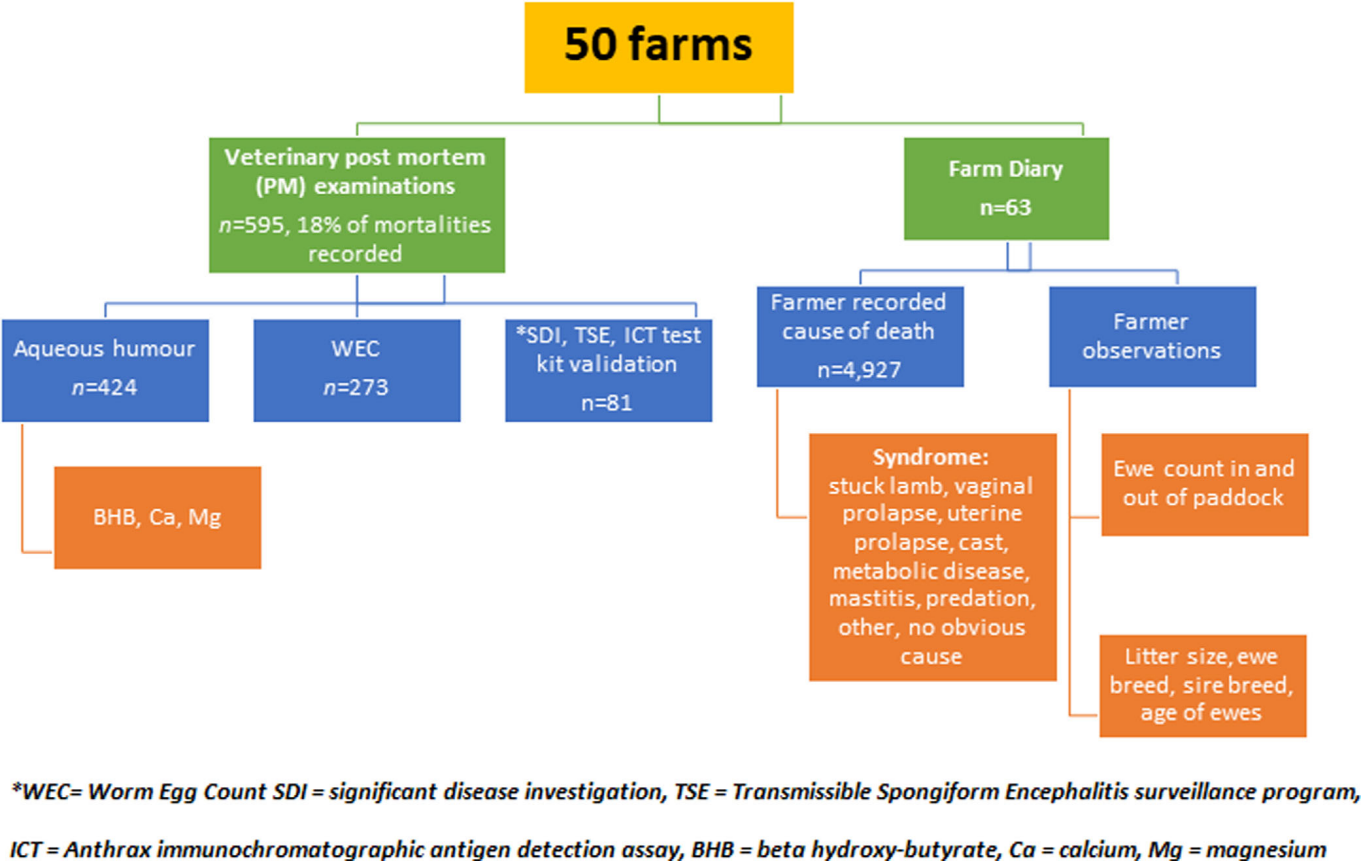
Summary of project methodology.

**Figure 2:**
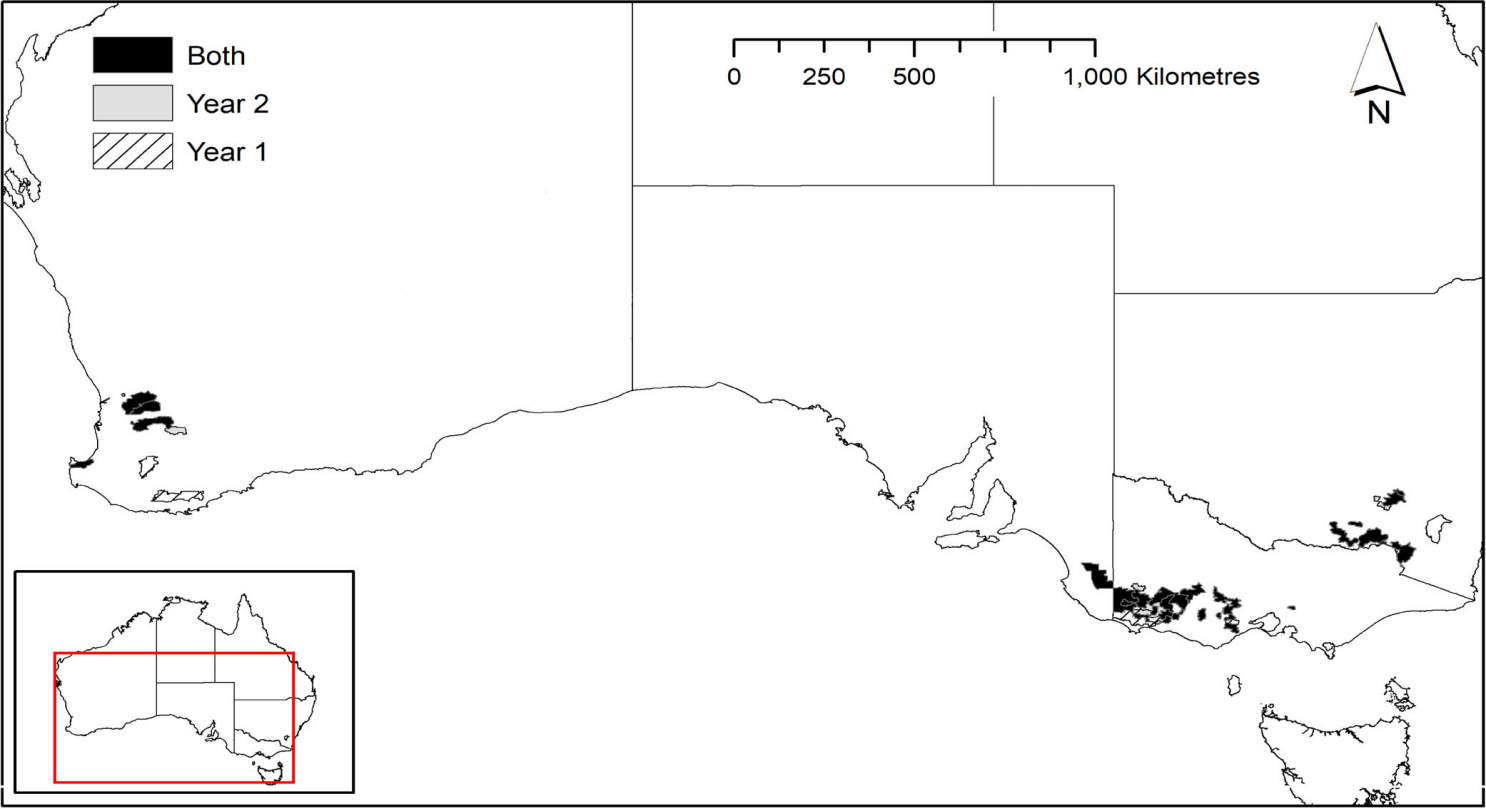
Postcodes of participating farms in Year 1 and Year 2.

A total of 50 commercial farms in southern Australia were included in the study (Figure 1), 30 farms participated in both years, 9 farms participated in Year 1 alone and 11 farms participated in Year 2 alone. Approximate location of farms is shown in Figure 2. Farms enrolled had a mean non‐Merino breeding ewe flock of 3544 ewes (95% CI 2930–4159). Overall, 223,297 breeding ewes were monitored, representing approximately 2% of the non‐Merino ewe population in Australia.^1^

In order to determine peri‐parturient ewe mortality and the associated causes, two sources of data were collected from the enrolled farms including farmer‐recorded causes of death and veterinary PM examinations (Figure 1). The study required no change to routine farm management.

### Farmer observations

For each participating farm, the entire non‐Merino breeding ewe flock was included in the project. Ewes were moved into their lambing paddocks and monitored in those paddocks until lamb marking (which occurred 1–4 weeks after all the ewes had lambed). A count of ewes placed into each lambing paddock was recorded by each farmer. Additional data relating to the paddock and the group of sheep in that paddock being recorded in a project designed farm diary including:

- Paddock name.
- Pregnancy ultrasound scanning category (singles/twins/triples/multiples/unscanned).
- Age of ewes at ram introduction or joining (tag colour if all one age or lambs (<1 year old) /hoggets (<2 years old)/2–5‐year‐old ewes/>5 years old).
- Ewe breed (First cross/ Composites (Romneys, Coopworths, Corriedales)/ British Breeds (Border Leicesters, White Suffolks)/Others (Dohne, Nudies, SAMMs, Dorpers)).
- Ram breed used for joining ewes (Poll dorset/ Composites (Romneys, Coopworths, Corriedales)/British Breeds (Hampshire, Border Leicester, White Suffolk)/ Others (Dohne, Nudie)).
- Pasture dry matter estimate when ewes were put into lambing paddocks (kg green/DM/ha) (estimated using a standardised Meat and Livestock Australia (MLA)) pasture ruler.^15^
- Pasture dry matter estimate at lamb marking (kg green/DM/ha) using the MLA pasture ruler as above.
- Number of ewes at lamb marking.
- Number of lambs at lamb marking.

From the time ewes were placed into their lambing paddocks up until lamb marking, farmers carried out routine lambing checks, which ranged from twice daily to twice weekly. Farmers did not record body condition score (BCS) at any time point in this study.

During these routine lambing checks, farmers recorded the number of ewe deaths, the date the ewe was found, as well as their suspected cause of death, which comprised allocation to one of the following categories based on their prior knowledge of the conditions:

- Dystocia (“lamb stuck”).
- Vaginal prolapse.
- Uterine prolapse.
- Cast.
- Mastitis.
- Predation.
- Metabolic disease.
- Other (explanation required).
- No obvious cause.

Farmers were asked to only record one suspected cause of death per ewe. At lamb marking, farmers completed their diaries by recording the number of ewes and lambs counted out of each lambing paddock. Interventions during lambing by the farmer (e.g. ewes assisted with dystocia) were not recorded.

### Farmer‐reported cause of ewe death

Peri‐parturient ewe mortality on each farm is represented as cumulative mortality (also termed mortality risk or finite mortality rate^16^). Cumulative mortality is defined as the proportion of individuals alive at the start of a period that die over that period.^16^ This was calculated as the number of ewe deaths reported by the farmer from the time ewes were placed in their lambing paddocks up until marking, as a percentage of the number of ewes that entered the lambing paddocks.

Causes of death are presented as the percentage of all dead ewes on each farm with each cause of death as identified by the farmer. The mean proportion of deaths due to each cause was calculated across all farms and is presented as the mean (95% CI) proportion of ewes on farm that were suspected to have died of that disease for each year of the project. Where possible comparisons were made between cause of death from ewes of different age, parity and litter size.

### Veterinary PM examinations

Three farm visits per year were scheduled during the lambing period for each farm. During each visit a veterinarian conducted PM examinations on all ewes that had died in the preceding 48 hours. If no suitable carcasses were present on the day of the scheduled visit, the visit was either rescheduled or cancelled if no suitable date could be arranged. In Year 1 these visits were scheduled to coincide with peak lambing as it was assumed that this coincided with a higher mortality rate per day.^9^ In Year 2, if two or more deaths occurred in a 48‐hour timespan but after allocation to lambing paddocks, participating farmers were encouraged to contact a project vet to arrange a PM visit. This change to the methodology in Year 2 was initiated to improve representation of all causes of ewe mortality over the peri‐parturient period, acknowledging that some causes, for example, metabolic disease and vaginal prolapse are more likely to occur in the prepartum period.^9^

A PM examination protocol was developed to ensure a consistent approach to diagnosis by veterinarians, to ensure consistent and appropriate data collection and to facilitate retrospective examination of the findings. The findings were captured electronically through a secure online application developed in CommCare (CommCare, Dimagi, 2021).

Where possible, the minimum samples taken per PM examination were aqueous humour and faeces. Each aqueous humour sample was collected via a 20‐gauge needle attached to a 3–5‐mL syringes and stored frozen in a 10‐mL serum tube. The aqueous humour metabolites analysed included calcium, magnesium and BHB. Faecal samples were stored in zip lock bags with the air squeezed out and refrigerated (2–8°C) for a maximum of 7 days before worm egg counts (WEC's) were analysed on each sample using the McMaster technique.^17^ If a notifiable disease was suspected or carcasses were eligible for government funded laboratory support, additional relevant samples (e.g. larval culture) were submitted to relevant state department animal health laboratories.

Postmortem findings were not reported to the farmers during data collection unless the results were deemed by the consulting veterinarian to be unusual or concerning (e.g. high *Haemonchus contortus* burden – this occurred in three cases). Findings at PM examination were recorded against individual ewe identification number and taken into account when recoding the final diagnosed cause of death.

Within the PM protocol, veterinarians were asked to record their primary diagnosis and differential diagnoses and rate their confidence in the diagnosis on a scale of 1 (very confident) to 5 (not confident). Within the initial data collection, each ewe could have multiple diagnoses recorded representing disease associated diagnosis. These diagnoses included:

- Primary dystocia.
- Secondary dystocia.
- Trauma (including uterine, uterine artery, bladder and abdominal muscle rupture).
- Septicaemia (including metritis and peritonitis).
- Uterine prolapse.
- Vaginal prolapse.
- Cast.
- Pneumonia.
- Foot disease (foot rot, foot abscess an scald).
- Gastrointestinal nematodes.
- Dorsal vaginal wall rupture (DVWR).
- Mastitis.
- Pregnancy toxaemia.
- Hypocalcaemia.
- Hypomagnesaemia.
- Mixed metabolic.
- Other (including polioencephalomalacia, neoplasia, phalaris staggers, emaciation, Johne's and arthritis).
- Unknown.

Each case was subsequently assessed by one veterinarian. A single derived cause of death was recorded for each case based on both the findings reported by the attending veterinarian and the laboratory results.

Dystocia was considered the primary diagnosis when, on PM examination a ewe presented with malpresentation, foetopelvic disproportion, uterine torsion, cervical dilation failure (ring womb) or uterine inertia (i.e. normal presentation at time of death with or without associated hypocalcaemia).

Trauma was considered the primary cause of death in cases of bladder rupture, uterine artery or uterine rupture and abdominal muscle rupture with or without the presence of concurrent conditions such as septicaemia and hypocalcaemia.

Septicaemia was considered the primary diagnosis in cases of metritis, placentitis, enteritis and enterotoxaemia in the absence of any other cause except for concurrent hypocalcaemia and hypomagnesemia. In cases of septicaemia with concurrent hypocalcaemia (n = 16) and/or hypomagnesaemia (n = 4) the diagnosis was recorded as septicaemia.

Hypocalcaemia was considered the primary diagnosis when a ewe had a calcium level below 1 mmol/L^18^ in the absence of any other PM findings.

A normal BHB level in aqueous humour is between 0 and 0.8 mmol/L.^19^ Primary pregnancy toxaemia (PPT) was diagnosed in ewes bearing two or more lambs with signs of undernutrition including low BCS, peritoneal fat necrosis and/or gelatinous bone marrow with PM findings consistent with pregnancy toxaemia including a pale, friable swollen liver and two or more lambs *in utero* where BHB levels were above 2 mmol/L as a BHB level >2 mmol/L is considered diagnostic, whereas BHB >0.8 mmol/L is only considered supportive.^20^ No single‐bearing ewes in this study had BHB levels above 2 mmol/L. Secondary pregnancy toxaemia (SPT) was diagnosed in ewes with signs of pregnancy toxaemia, BHB levels above 2 mmol/L and concurrent conditions such as vaginal prolapse or foot issues.

Hypomagnesaemia was diagnosed in the absence of any other conditions and a magnesium level below 0.75 mmol/L.^21^

Internal parasitism was considered as diagnosis when strongylid WEC's exceeded 2000 eggs per gram (epg) and/or government funded laboratory support allowed for larval culture to diagnose haemonchosis in the absence of any other PM findings.

A ewe was diagnosed with an unknown cause of death when the differential diagnoses were not corroborated by laboratory findings.

### Statistical analysis

Where possible, mean cumulative mortality and 95% upper and lower confidence intervals (CIs) were reported for ewes of different age and litter size.

A generalised linear model (negative binomial regression) was used for analysis with peri‐parturient ewe mortality as the outcome variable. The initial input variables included ewe age, litter size, ewe breed, ram breed, region, parity and the number of paddock checks with the final analysis input variables including litter size, the number of paddock checks and ewe age with farm considered as a random factor. As the generalised linear model coefficients are on a log scale, the coefficients were exponentiated to estimate likelihoods. The results focus on the relative risk (x‐times risk) of significant factors, with CIs also being exponentiated. Relative risk was calculated for statistically significant output variables (P < 0.05).

The source of the ewe death data used to calculate the overall mortality varied between participating farms based on the farmers perception of the most accurate records. For example, if farmers had been accurately recording each death in each paddock up until lamb marking but at marking this figure did not match the difference in the counts of ewes into and out of the paddock, then the total number of deaths recorded in the farm diary was used. If, however, farmers were unsure if they recorded each ewe death accurately but were sure of their counts into and out of paddocks, then the difference between the counts into and out of the paddock was used.

Farmer‐reported causes of death are presented, on a farm basis, as proportional mortality, defined as the proportion of deaths that are caused by a specific disease^19^ and represented as a percentage.

Veterinarian diagnosed causes of death are presented for all farms in each year not on a farm basis due to the lower numbers of data per farm. Proportional mortality was calculated using the single derived cause of death as a percentage of the total number of all ewes submitted for PM examination in that year.

## Results

Flock size ranged from 698 to 11,969 ewes. In Year 1, 39 properties were recruited from four states and in Year 2, 41 properties (Table 1).

**Table 1:** Summary of number and location of participating sheep enterprises

State	Number of properties (n) (n*) Year 1	Number of properties (n) (n*) Year 2
Victoria	26 (6)	27 (7)
New South Wales	4 (0)	5 (1)
South Australia	2 (0)	3 (1)
Western Australia	7 (3)	6 (2)
TOTAL	39 (9)	41 (11)

(n*) = the number of properties in only 1 year of study.

Of the 50 farms enrolled over the course of the study, five farms either did not scan ewes or did not separately manage scanned ewes. All these farms were in Western Australia in both years of the study.

In Year 1, 27 of the 39 enrolled producers provided information that allowed the overall ewe mortality to be calculated either in the form of diary death counts or the difference between the number of ewes counted in and out of the lambing paddocks within the study period. On four properties the diary ewe deaths matched the difference in ewe counts (81 ewe deaths out of 7929 ewes). The difference in ewe head count was used for 17 properties (2220 ewe deaths out of 73,460 ewes), the farm diary counts were used for four properties (281 ewe deaths out of 17,654 ewes) and a mixture of both head counts and farm diary deaths were used to calculate deaths on two of the properties (85 ewe deaths out of 3921 ewes).

In year 2, 37 of the 41 recruited properties provided sufficient information to allow overall mortality to be calculated. The farm diaries matched the head counts on nine properties (414 ewe deaths out of 27,893 ewes). The difference in ewe head count was used for nine properties (476 ewe deaths out of 22,052 ewes) and the farm diary counts were used for 19 properties (1370 ewe deaths out of 70,388 ewes).

### Peri‐parturient ewe mortality

The peri‐parturient ewe mortality was 2.5% in Year 1 (95% CI 1.9%, 3.1%) for the 27 farms that provided information and 2.0% in Year 2 (95% Cl 1.7%, 2.0%) for the 37 farms that provided information (Table 2). There was no significant difference in overall mortality between Year 1 and Year 2 (P = 0.89).

**Table 2:** Percentile ranges for mean peri‐parturient ewe mortality over peri‐parturient period

Percentile ranges	Peri‐parturient ewe mortality Year 1	Peri‐parturient ewe mortality Year 2
0 to 25th	0.5%–1.4%	0.8%–1.3%
Median	2.1%	1.9%
75th to 100th	3.4%–5.9%	2.3%–4.7%

In Years 1 and 2, the overall peri‐parturient ewe mortality for the lowest 25th percentile of farms was ≤1.4% and ≤1.3%, respectively. Mortality in the highest 25th percentile of farms was ≥3.4% in Year 1 and ≥2.3% in Year 2 (Table 2).

Across all farms, the mean peri‐parturient ewe mortality for each litter size recorded is detailed in Table 3. When compared with single‐bearing ewes, ewes bearing all other litter sizes had a statistically higher risk of dying (P = <0.001). Triplet‐bearing ewes had the highest risk of death with a 3‐fold (95% CI: 2.18, 4.28) risk when compared with single‐bearing ewes and multiple and twin‐bearing ewes having a 1.9 (95% CI: 1.52, 2.28) and 1.6‐fold (95% CI: 1.23, 1.95) higher risk of death respectively when compared with single‐bearing ewes. Twin‐bearing ewes had a statistically lower risk of dying (P = <0.001) compared with triplet‐bearing and nonscanned ewes (P = <0.05) with triplet‐bearing ewes having a 2‐fold (95% CI: 1.47, 2.66) higher risk of death and nonscanned ewes having a 1.4‐fold (95% CI: 1.02, 2.04) higher risk of death when compared with twin‐bearing ewes.

**Table 3:** Ewe mortality for ewes in different litter sizes in both years of the study (whole of project figures from allocation to lambing paddock to lamb marking)

Litter size	Number of deaths	Number of ewes in to lambing paddocks	Mean peri‐parturient ewe mortality^1^	Standard deviation
Single	938	66,658	1.2%^a^	0.2%
Twin	1256	56,417	1.9%^b^	0.2%
Multiple^2^	1778	65,734	2.6%^bc^	0.3%
Triplet plus^3^	232	4593	4.8%^d^	1.0%
Nonscanned (WA flocks)	723	29,895	2.4%^cd^	0.5%

^1^ Numbers with different letter superscripts are significantly different from each other.

^2^ Scanned with two or more foetuses.

^3^ Scanned with 3 or more foetuses.

By age group, older ewes (>5 years) were statistically (P < 0.05) at greater risk of dying compared with all other age groups (Table 4). Older ewes were 1.6 times more likely to die compared with both hoggets (95% CI: 1.23, 2.08) and ewes aged 2–5 years old (95% CI: 1.27, 1.92) and 2‐fold (95% CI: 1.49, 2.74) at higher risk of death when compared with ewe lambs.

**Table 4:** Mean peri‐parturient ewe mortality (%) for ewes in different age categories in both years of the study (whole of project figures from allocation to lambing paddock to lamb marking)

Age	Number of deaths	Number of ewes in to lambing paddocks	Mean peri‐parturient ewe mortality^1^	Standard deviation
Primiparous ewes				
Ewe lambs at joining (<1 year old)	228	13,619	1.6%^a^	0.4%
Hoggets at joining (<2 years old)	379	17,209	1.9%^ab^	0.2%
Multiparous ewes				
2–5 years old at joining	2788	117,232	2.4%^b^	0.2%
>5 years old at joining	321	9785	3.1%^c^	0.7%

^1^ Numbers with different letter superscripts are significantly different from each other.

Parity, ewe breed, ram breed and the number of paddock checks were not statistically significant risk factors for peri‐parturient ewe mortality in this study.

### Farmer‐reported cause of death

A total of 4927 ewe deaths were reported across both years of the study out of 223,297 ewes.

**Figure 3:**
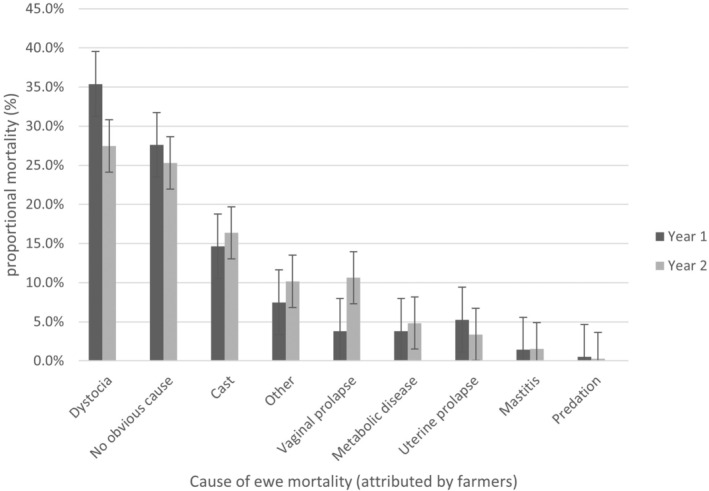
Farmer‐reported cause of death for Year 1 (n = 1311) and Year 2 (n = 2005) represented as the proportional mortality (%) attributed to each condition across participating farms. Error bars represent the upper and lower 95% CI for each cause of death.

Obvious dystocia (“lamb stuck”) was the most commonly recorded cause of death (30.6%) in both years of the study (Year 1, 35.4% and Year 2 27.5%) followed by “no obvious cause of death” in 26.2% of cases (Year 1, 27.6%; Year 2, 25.3%) (Figure 3).

The “other” category comprised 7.5% of reported deaths in year 1, and 10.2% of the cases in Year 2 including ewes being in poor condition, phalaris staggers, misadventure, euthanasia and trauma cases such as abdominal muscle rupture.

Metabolic disease was reported as the suspected cause of death in 3.8% of deaths reported in Year 1 and 4.8% of deaths reported in Year 2.

The risk of dystocia was associated with age at maiden lambing in both years (P = <0.01). In Year 1, ewes that first gave birth as ewe lambs were 2.6 times (95% CI: 1.68, 3.86) more likely to have an obvious dystocia recorded as cause of death compared with ewes that first gave birth as 2‐year‐old maidens. In Year 2, ewe lambs were 1.6 times (95% CI: 1.68, 3.86) more likely to have obvious dystocia recorded as a cause of death compared with 2‐year‐old maidens.

### Veterinary diagnosed cause of death

A total of 595 ewe PM examinations were performed over both years of this project (294 in Year 1, 301 in Year 2), representing 17% of the recorded mortalities in Year 1 and 18% in Year 2. Seventy‐three percent (432) of these cases had aqueous humour results.

**Figure 4:**
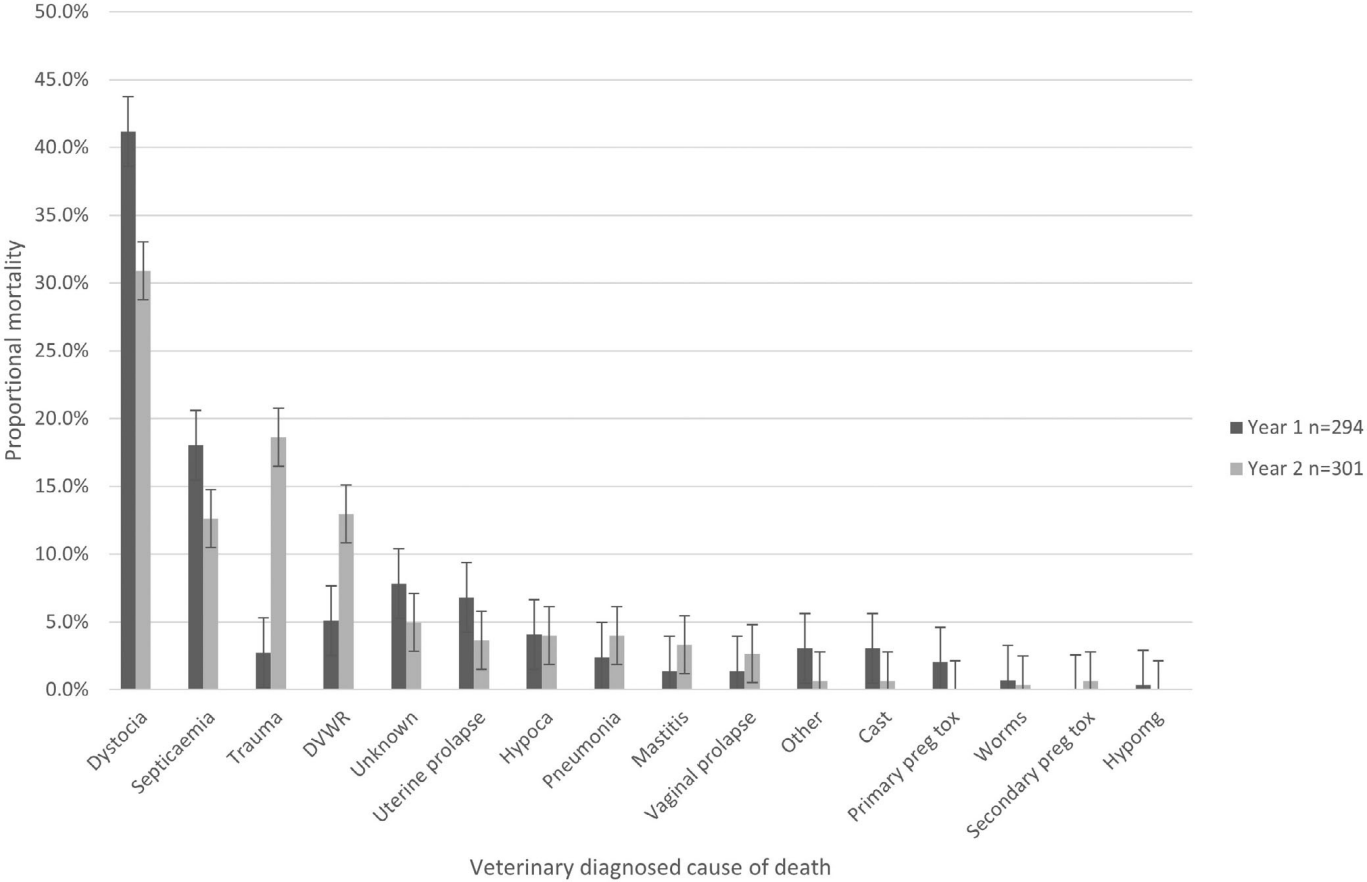
Percentage of cases with each veterinary diagnosed cause of death for Year 1 (294 PM examinations from 35 farmers) and Year 2 (301 PM examinations from 38 farmers). DVWR, Dorsal vaginal wall rupture.

The single derived cause of death diagnosed by veterinary PM in Year 1, Year 2 and combined are shown in Table 5. The most frequently diagnosed derived cause of death in both years was dystocia (Overall, 36%; Year 1, 41.2%; Year 2, 30.9%) (Figure 4).

**Table 5:** Description of the causes of death based on veterinary postmortem (PM) examination and ancillary diagnostics tests with associated diagnosis

Derived cause of death (n)^a^	Cause of death description (n)	Description of associated diagnoses	Mean BCS
Cast (11)	Cast only (3) Cast with associated diagnoses (8)	Hypocalcaemia (8)	2.9
DVWR (54)	DVWR only (37) DVWR with associated diagnosis (17)	Hypocalcaemia (6) Trauma (9) Septicaemia (3) Vaginal Prolapse (4)	3.3
Dystocia (218)	Dystocia only (62) Dystocia with associated diagnosis (156)	Elevated BHB (1) Hypocalcaemia (36) Hypomagnesaemia (4) Septicaemia (77) Trauma (111)	3.0
Hypocalcaemia (23)	Hypocalcaemia only (23)	—	2.5
Hypomagnesaemia (1)	Hypomagnesemia only (1)	—	‐
Internal Parasitism (3)	Internal Parasitism only (3)	—	2.6
Mastitis (16)	Mastitis only (8) Mastitis with associated diagnosis (8)	Hypocalcaemia (4) Hypomagnesaemia (1) Septicaemia (4)	2.8
Other (12)	Emaciation (2) Johne's (1) Neoplasia (3) Phalaris Staggers (2) Polioencephalomalacia (3) Polyarthritis (Erysipelothrix *rhusiopathiae*) (1)	—	2.5
Pneumonia (17)	Pneumonia only (7) Pneumonia with associated diagnosis (10)	Hypocalcaemia (8) Septicaemia (2)	2.9
Pregnancy Toxaemia (8)	Primary Pregnancy Toxaemia (PPT) (6) Secondary Pregnancy Toxaemia (SPT) (2)	Hypomagnesaemia (1)	PPT 2.6 SPT 3.4
Septicaemia (87)	Septicaemia only (68) Septicaemia with associated diagnosis (19)	Hypocalcaemia (16) Hypomagnesaemia (4)	2.7
Trauma (63)	Trauma only (19) Trauma with associated diagnosis (44)	Elevated BHB (3) Hypocalcaemia (8) Septicaemia (28)	2.9
Unknown (39)	Unknown at PM examination (31) Unknown (findings not corroborated) (8)	—	2.6
Uterine Prolapse (31)	Uterine prolapse only (25) Uterine Prolapse with associated diagnosis (6)	Hypocalcaemia (3) Hypomagnesaemia (2) Flystrike (1) Septicaemia (1)	3.0
Vaginal prolapse (12)	Vaginal prolapse with associated diagnosis (12)	Hypocalcaemia (6) Hypomagnesaemia (2) Septicaemia (4) Trauma (7)	3.0

^a^ Multiple associated diagnosis could be recorded for each individual case in initial data collection phase.

(n) = number of ewes.

DVWR, Dorsal vaginal wall rupture.

External evidence of dystocia before PM examination (e.g. protruding fetal head/s or limb/s) was only apparent in 66% of the 121 dystocia cases in Year 1 (95% CI: 56%, 75%) and 57% of the 97 cases of dystocia in Year 2 (95% CI: 46%, 67%) and were reported as “obvious dystocia.”

Of the cases diagnosed with dystocia, signs of trauma (either uterine, uterine artery or bladder rupture) were present in 36.4% in Year 1 and 71.0% in Year 2.

Septicaemia was determined to be the primary cause of death in 15.3% of cases overall (Year 1, 18.0% and Year 2, 12.6%). Trauma was diagnosed in 10.8% of cases over both years but at a much lower rate in Year 1 (2.7%) compared with Year 2 (18.6%), as was DVWR(5.1% and 13.0%, respectively).

“Other” causes of death diagnosed accounted for 1.0% of the PM examinations in both years (Year 1, 3.1% and Year 2, 0.8%).

Unknown causes of death accounted for 6.4% of PM examinations overall (Year 1, 7.8% and Year 2, 5%).

The mean BCS for ewes submitted for PM examination in both years was 2.9 (95% CI 2.5, 3.25). In Year 1, the deceased ewes had a statistically lower (P = <0.001) mean BCS of 2.7 (95% CI 2.5, 3.25) compared with 3.0 in Year 2 (95% CI 2.75, 3.5).

## Discussion

Mean non‐Merino peri‐parturient ewe mortality was 2.5% (Year 1) and 2.0% (Year 2). This represents the first estimate of peri‐parturient ewe mortality in non‐Merino populations in Australia. Previous estimates have not delineated between Merino and non‐Merino ewes and /or have been based on annual mortality levels.^4^, ^5^, ^6^, ^7^, ^8^ A peri‐parturient mortality level between 2% and 2.5% is consistent with the annual rates of 2% and 10% reported in Australia^4^, ^5^, ^6^, ^7^, ^8^; however, given that annual mortality rates were not measured in this study, we cannot conclude whether the observed death rate was higher in the observation time period when compared with other periods of the year. Hence, we cannot confirm the sentiments by Mavrogianni and Brozos (2008) that ewe mortality is most likely to occur during the peri‐parturient period.

There was considerable variation between mobs on farms (0%–16.7%), as well as between farms (0.5%–5.9%) as would be expected given the variable mortality risk profiles. This variation suggests that, even among farmers who have largely adopted LTEM guidelines (including practices like pregnancy scanning and differential management), there may still be opportunities for further improvement.

In terms of litter size, the mortality rate for ewes categorised as multiple bearing was 2.6%. Interestingly, if the multiple‐bearing group had the same proportion of twins and triplets as the groups managed separately for twins and triplets, and the same differential mortality rates, the overall mortality would be 2.7%, matching the mortality rate observed in the ewes managed as multiple bearing.

Farmers reported dystocia to be the most common cause of peri‐parturient ewe death (30.6% of the deaths in both years). This aligned with the veterinary diagnoses based on PM examination that identified dystocia in 36.1% ewes submitted for PM examinations. However, without veterinary PM examination, dystocia cases were likely to be underreported by producers, with indicators of “obvious dystocia” evident in only 66% and 57% of PM examinations in Years 1 and 2, respectively. It is likely that the overall risk of dystocia related death was underestimated in this study as cases of trauma related death (including bladder rupture, uterine artery or uterine rupture and abdominal muscle rupture) and cases of septicaemia (including metritis and placentitis) are possibly associated diagnoses to dystocia. Based on these figures, the real incidence of dystocia on the surveyed farms may have exceeded 50% of observed peri‐parturient ewe deaths. Dystocia being the leading cause of ewe mortality was consistent with Australian studies where dystocia was a major contributor to perinatal lamb mortality.^22^

Farmers reported unknown cause of death in 26.2% of cases, whereas only 6.4% of ewes that underwent a veterinary PM had an unknown cause of death. Therefore, although farmer‐recorded cause of death data may be a useful tool to understand the cause of peri‐parturient death for conditions with obvious external signs, such as some dystocia cases, other conditions need PM examination and/or further ancillary tests to determine the cause of death. As such, conditions such as dystocia, metritis and metabolic conditions such as hypocalcaemia may go underreported.

Trauma was diagnosed by veterinarians at a much lower rate in Year 1 (2.7%) compared with Year 2 (18.6%), as was DVWR (5.1% and 13.0%, respectively). Both of these conditions are more likely to occur in ewes with high BCS^23^ and given ewes that underwent PM examinations had a statistically lower mean BCS in Year 1 compared with Year 2 (2.7 and 3.0 respectively), it is possible that the increases in trauma in Year 2 was related to ewes being in better condition score.

Farmer paddock checks ranged from twice daily to twice weekly, but no significant association was found between the frequency of these checks and ewe mortality. This suggests that simply increasing the frequency of checks may not be sufficient to reduce mortality rates. It is possible that although some gains might be made by assisting ewes for example in cases of dystocia or being cast, these could be offset by the stress or complications introduced by the presence of the farmer or their attempts to isolate and capture ewes in distress. Such interventions might inadvertently lead to additional difficulties or mortalities in other ewes. Further research is warranted to assess the effectiveness of these paddock checks in preventing ewe mortality and to explore whether the intervention methods used during these checks could be refined to minimise potential negative impacts. Additionally, the number of ewes assisted and/or saved by farmers during lambing inspections was not recorded in this study, which may mean that ewe mortality could have been higher than recorded. However, the extent of this potential difference could not be determined.

Triplet‐bearing ewes had a greater risk of death compared with single‐bearing, twin‐bearing and unscanned ewes. This is in keeping with Australian and international studies.^6^, ^8^, ^10^ The mortality in triplet‐bearing ewes in this study (4.8%) was comparable with that reported for non‐Merino ewes (4.9%) and Merino ewes (6.7%) in a survey of 95 Australian producers.^24^

Ewes older than 5 years were at greater risk of death compared with all other ages groups including ewe joined as lambs, maiden hoggets and multiparous ewes 2–5 years old. Current recommendations for ewe management are based on data derived for mixed age ewes^25^, ^26^ and therefore refinement of recommendations specific to older ewes to maintain productivity and welfare over lambing for this age group is warranted if retention of older ewes is used as a strategy to rebuild flock numbers. Brien et al. (2023) identified the retention of older ewes as one of the top strategies for rapidly and profitably rebuilding flock numbers in Australia's sheep industry.^27^ However, the study also emphasised the importance of tailored management practices to mitigate the increased mortality risk in older ewes. These practices include enhanced nutritional support to maintain body condition, particularly during critical periods such as late pregnancy and lambing, and more frequent health monitoring to promptly address any emerging issues. Additionally, the study suggested that separating older ewes from younger flocks to manage them more intensively could further help in reducing mortality and maintaining their productivity.^27^

Metabolic diseases were not commonly identified as a primary cause of death by either farmers or through the single derived cause of death in veterinary PM examinations. For farmer‐reported data, this may be due to the difficulty in differentiating metabolic diseases from other causes of death without further diagnostics, such as aqueous humour evaluation.

The use of a single derived cause of death in veterinary PM examinations limits the identification of metabolic diseases, which may often be a contributing factor rather than the primary cause. This method, aimed at mirroring farmer‐reported causes of death, could lead to an underreporting of metabolic diseases. Moreover, veterinarians in this study conducted PM examinations primarily during the peak of lambing especially in Year 1 of the study. Metabolic diseases like hypocalcaemia and pregnancy toxaemia, however, are more likely to occur prepartum, potentially leading to their underrepresentation in the PM findings.

There was no significant association between ewe or ram breed with peri‐parturient ewe mortality, and therefore, these factors were not included in the final generalised linear model. However, breeding values for ewes and sires of lambs were not available and further investigation of the relationship between genetic traits and ewe survival such as birth weight and lambing ease are warranted given dystocia was a leading cause of ewe mortality.

Parity also was not included in the final generalised model as it was not a significant risk factor in this study; however, categories were only split between first parity and second or more which may have diluted the effects of this factor.

The study population consisted of commercial, non‐Merino ewes in southern Australia. According to a fundamental principle of research and scientific theory, results from observational studies are typically generalised only to the study population, serving primarily as a basis for generating hypotheses about disease behaviour in the broader population. However, this study identified risk factors for mortality, such as ewe age and litter size, which are intrinsically linked to the disease processes of significant conditions observed, including dystocia, sepsis and trauma. Although these risk factors are likely applicable to non‐Merino ewes more broadly, the strength of these associations may differ in other populations. Therefore, when making practical decisions about disease control, these findings may still be relevant under similar management and ecological conditions, although their generalisability should be considered with caution.

## Conclusion

This is the first study to determine the level of peri‐parturient mortality in non‐Merino ewes in commercial flocks in Australia. Dystocia, septicaemia and trauma were important causes of ewe mortality over the peri‐parturient period. Strategies to reduce peri‐parturient ewe mortality include managing ewes carrying twins or triplets and ewes aged 5 years or older to ensure nutritional targets are met while integrating management and selection strategies aimed at reducing dystocia.

## Ethics statement

Animal Ethics Committee (AEC) approval was sought in all states involved in the project. The following committees were consulted: the Secretary's Animal Care and Ethics Committee (NSW), the Wildlife and Small Institutions AEC (Vic), the Department of Primary Industries and Regions AEC (SA) and the Department of Primary Industries and Regional Development AEC (WA). It was concluded by these committees that, as the project involved no intervention to normal management practices and ewes were not euthanased for research purposes, the project did not fall within the definition of “animal research” under the Animal Research Act 1985 and, therefore, did not require AEC approval.

## Conflicts of interest and sources of funding

None of the authors have a conflict of interest to disclose.

## Data Availability

The data that support the findings of this study are available from Meat & Livestock Australia. Restrictions apply to the availability of these data, which were used under license for this study. Data are available from the author(s) with the permission of Meat & Livestock Australia.
